# A New Medical Device to Provide Independent Ventilation to Two Subjects Using a Single Ventilator: Evaluation in Lung-Healthy Pigs

**DOI:** 10.1155/2020/8866806

**Published:** 2020-12-30

**Authors:** Ignacio Lugones, Roberto Orofino Giambastiani, Oscar Robledo, Martín Marcos, Javier Mouly, Agustín Gallo, Verónica Laulhé, María Fernanda Biancolini

**Affiliations:** ^1^Hospital General de Niños “Pedro de Elizalde”, Ciudad Autónoma de Buenos Aires, Buenos Aires, Argentina; ^2^Facultad de Ciencias Veterinarias, Universidad Nacional de La Plata, Provincia de Buenos Aires, La Plata, Argentina

## Abstract

**Background:**

The global crisis situation caused by SARS-CoV-2 has created an explosive demand for ventilators, which cannot be met even in developed countries. Designing a simple and inexpensive device with the ability to increase the number of patients that can be connected to existing ventilators would have a major impact on the number of lives that could be saved. We conducted a study to determine whether two pigs with significant differences in size and weight could be ventilated simultaneously using a single ventilator connected to a new medical device called DuplicARⓇ.

**Methods:**

Six pigs (median weight 12 kg, range 9–25 kg) were connected in pairs to a single ventilator using the new device for 6 hours. Both the ventilator and the device were manipulated throughout the experiment according to the needs of each animal. Tidal volume and positive end-expiratory pressure were individually controlled with the device. Primary and secondary outcome variables were defined to assess ventilation and hemodynamics in all animals throughout the experiment.

**Results:**

Median difference in weight between the animals of each pair was 67% (range: 11–108). All animals could be successfully oxygenated and ventilated for 6 hours through manipulation of the ventilator and the DuplicARⓇ device, despite significant discrepancies in body size and weight. Mean PaCO_2_ in arterial blood was 42.1 ± 4.4 mmHg, mean PaO_2_ was 162.8 ± 46.8 mmHg, and mean oxygen saturation was 98 ± 1.3%. End-tidal CO_2_ values showed no statistically significant difference among subjects of each pair. Mean difference in arterial PaCO_2_ measured at the same time in both animals of each pair was 4.8 ± 3 mmHg, reflecting the ability of the device to ventilate each animal according to its particular requirements. Independent management of PEEP was achieved by manipulation of the device controllers.

**Conclusion:**

It is possible to ventilate two lung-healthy animals with a single ventilator according to each one's needs through manipulation of both the ventilator and the DuplicARⓇ device. This gives this device the potential to expand local ventilators surge capacity during disasters or pandemics until emergency supplies can be delivered from central stockpiles.

## 1. Background

As a consequence of sudden major catastrophes such as terrorism attacks, natural disasters, massive accidents, or epidemic outbreaks, health systems have focused on the need for simultaneous medical attention for a large number of victims [1–3].

Depending on the nature and suddenness of the disaster, many hospital supplies, such as ventilators, may become insufficient to face the demand. Although health system resources can be immediately redirected for equipment provision, there is a variable time period between the initial need of supplies and the moment they can actually be provided. During this interval, many patients could die [4, 5].

The world crisis triggered by the SARS-CoV-2 pandemic has created an explosive demand for mechanical ventilators. Such demand could not be met even in developed countries [6]. Healthcare teams are pushed to decide whether to withdraw or withhold ventilators for some patients and use them for other patients who have better chances of survival. This scenario requires the preparation of a triage system to better allocate the critical care resources available to maximize the benefit for the greatest number of people [7], which could create a possible legal liability [8].

The design of a simple and inexpensive device that allows simultaneous and independent ventilation of two patients with only one ventilator could have a significant impact on the number of lives that could be saved.

The objective of this study is to test, in a lung-healthy porcine model, a device designed to ventilate two subjects with the same ventilator, called DuplicARⓇ (patent application submitted).

## 2. Materials and Methods

### 2.1. Protocol Design

This is a translational research study with an experimental prospective and controlled protocol design, approved by the Institutional Animal Care and Use Committee of the School of Veterinary Sciences, National University of La Plata, Argentina, and the Ethics Committee of the General Children's Hospital of Buenos Aires “Dr. Pedro de Elizalde,” Argentina.

### 2.2. Animal Model

Six healthy pigs, with variable weights (median weight 12 kg, range 9–25 kg), coming from the local farm of the School of Veterinary Sciences of the National University of La Plata, previously dewormed and free of contagious infectious diseases, were included in the study. Animal handling was carried out following an update of the Guide for the Care and Use of Laboratory Animals of the National Research Council US Committee [9].

### 2.3. Preparation and Anesthesia

After intramuscular sedation with midazolam 0.5 mg/kg (Richmond Vet Pharma, Buenos Aires, Argentina) and ketamine 15 mg/kg (Richmond Vet Pharma, Buenos Aires, Argentina), each animal was connected to pulse oximetry and cardiac leads monitoring and placed in a prone position to allow ear vein cannulation. A surgical plane of anesthesia was obtained with intravenous propofol at 8 mg/kg in an induction dose and 15 mg/kg/h in continuous infusion (Richmond Vet Pharma, Buenos Aires, Argentina). The animals were intubated orotracheally and ventilated manually with 100% oxygen throughout the setup phase. The tibial artery was cannulated with a 20-gauge single lumen catheter, used for blood pressure measurements and arterial blood gas sampling. Mean arterial blood pressure (MAP), heart rate (HR), and pulse oximetry (SpO_2_) were recorded using multiparameter monitors (PM-9000, Shenzhen Mindray Bio-Medical Electronics, Shenzhen, China).

At this time, the animals were connected to the ventilator after an intravenous dose of vecuronium bromide 0.1 mg/kg (Scott-Cassará, Buenos Aires, Argentina).

### 2.4. The DuplicARⓇ Device

**Figure 1:**
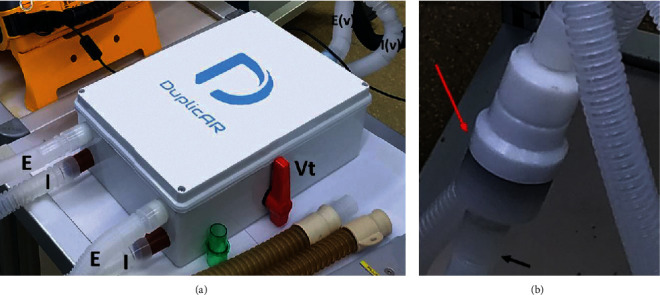
The DuplicARⓇ device: (a) the device with its connections; (b) the PEEP valve controller. E, expiratory limb of each subject; E(v), expiratory limb connected to the ventilator; I, inspiratory limb of each subject; I(v), inspiratory limb connected to the ventilator; Vt, tidal volume/peak-pressure controller; red arrow, PEEP controller; black arrows, expiratory limb.

The animals were connected in pairs, each pair to a single ventilator using the new device. DuplicARⓇ has two parts integrated in the same device (Figure 1).

The inspiratory part of the device connects the inspiratory port of the ventilator to each animal's inspiratory limb. It has ball valves that regulate flow (and therefore tidal volume (*V*_T_) and peak pressure) and one-way valves that (1) allow for independent management of the two circuits and (2) avoid cross-contamination.

The expiratory limb of each animal connects to the expiratory part of DuplicARⓇ, which in turn attaches to the expiratory port of the ventilator. It has one-way valves to prevent cross-ventilation and keep the patients' circuits isolated from each other. It also has a positive end-expiratory pressure (PEEP) valve, which is embedded in a small box and interposed in the expiratory limb of the patient that needs higher PEEP (Figure 1(b)). The amount of pressure configured on the PEEP valve will be added in that particular subject to the PEEP configured in the ventilator, in order to achieve the target PEEP for that subject. The remaining subject, with no PEEP valve interposed in the expiratory limb, will have the PEEP that was configured on the ventilator.

### 2.5. Ventilation

Volume assist-control ventilation was adjusted on the ventilator to achieve a *V*_T_ of 10–15 ml/kg of combined pig weight, respiratory rate between 16 and 22 breaths/min, inspiratory time between 0.75 and 1.3 seconds, fraction of inspired oxygen of 0.3, and PEEP of 2 cmH_2_0. The *V*_T_ controllers of the DuplicARⓇ device were manipulated to ensure adequate thoracic expansion and symmetrical pulmonary auscultation in both animals, according to their respective needs.

SpO_2_ and end-tidal CO_2_ (etCO_2_) were monitored continuously and provided important information during the initial ventilation setup. The airway pressure-time waveform of each animal was obtained using a regular arterial/venous pressure transducer (filled with air) connected to the *Y*-piece located at the confluence of the inspiratory and expiratory limbs of each subject and displayed in cmH_2_0 in the screen of the multiparameter monitor.

The animals were ventilated for 6 hours. A different ventilator was used in each experiment to test their compatibility with DuplicARⓇ: (a) Newport Breeze E-150 (Newport Medical Instruments, CA, USA), (b) Avea (CareFusion, CA, USA), and (c) Maquet Servo I (Maquet Critical Care AB, SE). Throughout the procedure, adjustments on the ventilator and the device were made based on clinical evaluation of each animal, cardiac monitoring, SpO_2_, capnography, and arterial blood gas parameters. In order to specifically assess the ability of the device to ventilate two different subjects independently, we focused on monitoring PaCO2, establishing the goal of achieving a normal value of this variable in arterial blood.

After 6 hours of combined ventilation, the different controllers of this novel device were manipulated in order to simulate the need for differential PEEP among animals. We performed selective increases in the PEEP in one animal of each pair through manipulation of the respective controller, in order to achieve a PEEP of 10 and 15 cmH_2_O sequentially, while maintaining a PEEP of 2 cmH_2_O in the other animal.

### 2.6. Data Collection and Analysis

Primary outcome variables reflecting ventilation and oxygenation performance were PaCO_2_, etCO_2_, PaO_2_, and SpO_2_. Secondary outcome variables were also defined: pH, MAP, HR, and serum lactate. Baseline (*t* = 0) was defined as the moment when both subjects were connected to the ventilator with the DuplicARⓇ device. Thereafter, vital signs were recorded every 15 minutes during the first hour, every 30 minutes during hours 2 and 3, and every 60 minutes between hours 4 and 6. Arterial blood samples were obtained at *t* = 30 min, *t* = 2 hr, and *t* = 5 hr and examined in a blood gas/electrolyte analyzer (Gem Premier 3000, Instrumentation Laboratory, Lexington, MA).

Primary and secondary outcome variables are reported as mean ± standard deviation and calculated using Microsoft Excel version X (Microsoft, Redmond, Washington, USA). Student's *t*-test was used for paired samples.

## 3. Results

Median difference in weight between the animals in each pair was 67%, ranging from 11% in the first experience to 108% in the last one.

**Figure 2:**
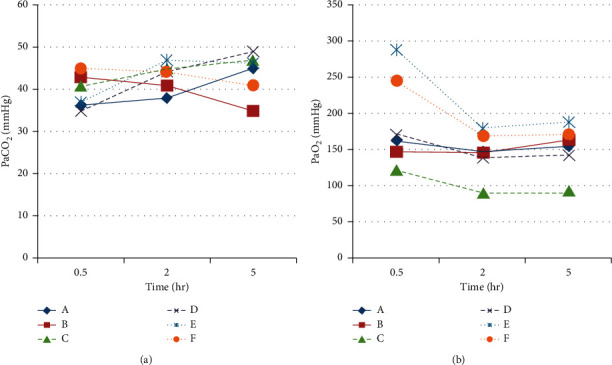
(a) PaCO_2_ and (b) PaO_2_ registered at *t* = 30 minutes, 2 hr, and 5 hr in all subjects.

Mean PaCO_2_ in arterial blood was 42.1 ± 4.4 mmHg (Figure 2). No statistically significant difference was found in this variable between subjects connected to the same ventilator at any time of the experiment. Mean difference in PaCO_2_, measured at the same time in both animals, in each pair was 4.8 ± 3 mmHg. An adequate oxygenation was maintained throughout the experiment in all animals, as reflected by a mean PaO_2_ of 162.8 ± 46.8 mmHg, while mean SpO_2_ was 98 ± 1.3%.

EtCO_2_ values were registered hourly and compared between animals. No statistically significant difference was found between subjects connected to the same ventilator (A-B pair: *p*=0.65; C-D pair: *p*=0.64; E-F pair: *p*=0.73).

All subjects remained hemodynamically stable during the procedure. MAP was 79 ± 17 mmHg, while mean HR was 115 ± 27 beats/min. Mean pH was 7.46 ± 0.05, and serum lactate values were below 1.31 mmol/l in all animals at all times (Table 1).

**Figure 3:**
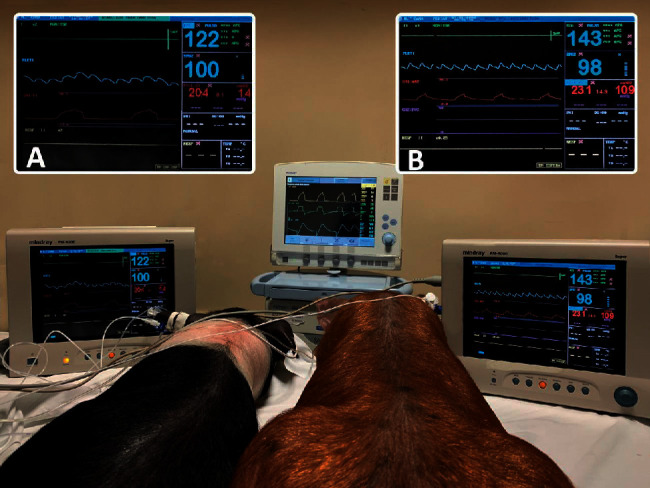
Two animals, weighing 12 kg (left) and 25 kg (right), are shown being ventilated simultaneously with a single ventilator and the DuplicARⓇ device. The pressure-time curve of each animal is displayed in cmH_2_O in each multiparameter monitor (in detail in (a) and (b)). In (a), the peak pressure is 20.4 cmH_2_O, while the PEEP is 1.4 cmH_2_0. In (b), the peak pressure is 23.1 cmH_2_0, and the PEEP is 10.9 cmH_2_0.

Manipulation of the PEEP controllers in one animal of each pair (animals A, C, and E) allowed a selective increase of the PEEP in that animal to 10 and 15 cmH_2_O successively, while PEEP in the other animal remained around 2 cmH_2_O (as set in the baseline configuration of the ventilator) (Figure 3).

## 4. Discussion

The idea of ventilating multiple patients with a single mechanical ventilator was described by Neyman and Irvin in 2006 [10]. In this study, four lung simulators were ventilated with a single ventilator, using 3-way connectors to have 4 inspiratory limbs and 4 expiratory limbs. In 2008, Paladino and collaborators tested the proposed method in an animal model, concluding that it was feasible to simultaneously ventilate four sheep with a single ventilator for 12 hours [11]. Afterwards, Smith and Brown reported simultaneous ventilation of two healthy human beings with a single ventilator and emphasized on the importance of matching both subjects such that both could tolerate the chosen pressures and achieve an acceptable *V*_T_ [12]. However, in 2012, Branson and Rubinson concluded that it was not possible to control the *V*_T_ that each patient would receive and the disparity in this volume was due to the variability in the airway compliance between patients [13]. Also, the pressure control mode exacerbated the disparity more than the volume-control mode.

During the COVID-19 pandemic, several healthcare teams have considered multiple ventilation (using 3-ways connectors in the inspiratory and expiratory ports of the ventilator) as an attractive and rapidly available alternative. However, this strategy has many limitations that make it an unsafe option. In fact, it has been totally discouraged by some of the most important scientific societies in the world [14]. The main drawback of this mode is its inability to manage the ventilatory parameters independently for each subject, especially the *V*_T_, the peak pressure, and the PEEP, both in the initial connection and over time. The lack of a device that distributes pressure and flow according to the needs of each subject implies that the subjects must meet similar criteria in terms of weight, clinical condition, and pulmonary compliance to become candidates to be ventilated simultaneously [15]. On the other hand, there are important challenges in monitoring ventilation and setting alarms, and it is necessary to consider that a sudden event occurring in one patient (e.g., disconnection, pneumothorax, and obstruction of the endotracheal tube) will impact on the other.

As part of the COVID-19 surge response plan, many groups have explored alternative strategies to overcome the problems of ventilator sharing [16]. At the beginning of the pandemic, our group developed a device called DuplicARⓇ as part of the efforts to face the disease in our country. In this pilot study, we show that it is possible to ventilate two lung-healthy subjects connected simultaneously to one ventilator and this novel device, according to their particular needs. Our first prototype was able to accomplish its main purpose, which was to achieve individual management of the *V*_T_ (or peak pressure) and the PEEP in response to the requirements of each subject over time. All the animals were successfully ventilated and oxygenated for 6 hours and were hemodynamically stable all along the experiment. The targeted values of PaCO_2_ and etCO_2_, the main endpoints that reflected ventilation, remained within expected ranges in all cases, and most importantly, there were no significant differences in these variables between animals connected to the same ventilator despite significant discrepancies in body size and weight. This is the reason why we prefer to call this strategy “combined ventilation” rather than “multiple ventilation,” as mutual interactions between the involved subjects are considered and individual needs are attended in order to ventilate them adequately. Regarding ventilation mode, pressure-controlled is probably safer than volume-controlled ventilation, as the maldistribution of the *V*_T_ in the latter can cause an excessively high *V*_T_ to be delivered to one individual, which can result in volutrauma and worse situations, including barotrauma.

This early-stage prototype is under continuous improvement. At this moment, the pressure-time curve can be clearly displayed in each subject's multiparameter monitor using a pressure transducer and setting the monitor to measure in cmH_2_O. This waveform allows for precise monitoring of peak pressure, plateau pressure and PEEP, the evaluation of the adequacy of the inspiratory flow, and the presence of auto PEEP, dynamic hyperinflation, or circuit leaks. The final version of the device will include direct real-time measurement of all variables of each subject and electronic closed loop control of peak pressure and PEEP of each individual through a human-machine interface.

It is important to note that this device is conceived to function only as a bridge to other alternatives in the context of disaster surge, when health systems are overwhelmed, and there are not as many ventilators as needed. To face these catastrophic events, this device can be easily stored and may provide fast availability of ventilation alternatives. As evidenced by this experiment, DuplicARⓇ is compatible with the three different ventilators that were used and would probably adapt well to other models.

Specific recommendations for optimal use of this device are as follows: (a) subjects need to be appropriately sedated and paralyzed to prevent patient-ventilator interaction; (b) individual airway pressure measurement should be provided in multiparameter monitors; (b) pressure limit and alarms must be carefully set to avoid excessive peak pressure; (c) closed aspiration circuits should be ensured to prevent contamination and need for disconnection; (d) filters must be used in both inspiratory and expiratory limbs; and (e) capnography and respiratory mechanics monitoring are highly recommended for each patient.

### 4.1. Study Limitations

This is just a proof-of-concept experiment, in which we only demonstrate that two subjects can be ventilated independently with one ventilator using DuplicARⓇ. The sample is too small to achieve strong statistically significant conclusions.

The device allows independent manipulation of *V*_T_ (or peak pressure) and PEEP, but cannot control each subject's fraction of inspired oxygen, respiratory rate, and inspiratory time. Besides, this first prototype does not display (in the device itself) the curves and values of the different variables of both subjects nor does it have incorporated alarms.

A detailed evaluation of the performance of this device has been carried out both in a computational model and in the respiratory laboratory with two lung simulators, measuring all the relevant variables in different scenarios. Both experiments are being reported at the time this article is being written. The device has not yet been tested in subjects with lung injury, which is an important limitation.

Finally, the device must be tested in a pressure-controlled ventilation mode in an animal model. This mode is probably the safest to ventilate simultaneously two patients with the same ventilator [17].

## 5. Conclusion

It is possible to ventilate two lung-healthy animal subjects with the same ventilator using DuplicARⓇ to regulate the *V*_T_ and the PEEP independently. This strategy might evolve into a life-saving bridge alternative to palliate the consequences of the sudden shortage of ventilators during catastrophic events.

## Figures and Tables

**Table 1:** Primary and secondary outcome variables in the six animals throughout the experiment.

	Pair 1				Pair 2				Pair 3			
	*t* = 30′		*t* = 5 hr		*t* = 30′		*t* = 5 hr		*t* = 30′		*t* = 5 hr	
	A	B	A	B	C	D	C	D	E	F	E	F
Weight	10	9			20	12			25	12		
pH	7.46	7.4	7.37	7.46	7.54	7.58	7.47	7.45	7.57	7.48	7.46	7.5
PaCO_2_	36	43	45	35	41	35	47	49	37	45	46	41
PaO_2_	162	148	156	164	123	171	94	143	289	246	189	172
SO_2_	99	99	99	100	96	99	95	99	99	100	96	100
HR	116	120	101	128	108	101	124	90	119	90	158	106
MAP	80	70	68	80	138	89	93	70	90	88	103	92
Lactate	1.24	0.96	1.29	0.87	0.5	0.9	0.5	0.8	0.7	0.8	0.6	1

## Data Availability

The data used to support the findings of this study are available from the corresponding author upon request.
